# The CD44^+^/CD24^- ^phenotype is enriched in basal-like breast tumors

**DOI:** 10.1186/bcr2108

**Published:** 2008-06-17

**Authors:** Gabriella Honeth, Pär-Ola Bendahl, Markus Ringnér, Lao H Saal, Sofia K Gruvberger-Saal, Kristina Lövgren, Dorthe Grabau, Mårten Fernö, Åke Borg, Cecilia Hegardt

**Affiliations:** 1 Department of Oncology, Clinical Sciences, Lund University, Barngatan 2B, SE-221 85 Lund, Sweden; 2 CREATE Health Strategic Centre for Clinical Cancer Research, Klinikgatan 28, SE-221 85 Lund, Sweden; 3 Institute for Cancer Genetics, Columbia University, 1150 St Nicholas Ave, New York, NY 10032, USA; 4 Department of Pathology, Clinical Sciences, Lund University, Sölvegatan 25, SE-221 85 Lund, Sweden; 5 Lund Strategic Research Center for Stem Cell Biology and Cell Therapy, Klinikgatan 26, SE-221 85 Lund, Sweden

## Abstract

**Introduction:**

Human breast tumors are heterogeneous and consist of phenotypically diverse cells. Breast cancer cells with a CD44^+^/CD24^- ^phenotype have been suggested to have tumor-initiating properties with stem cell-like and invasive features, although it is unclear whether their presence within a tumor has clinical implications. There is also a large heterogeneity between tumors, illustrated by reproducible stratification into various subtypes based on gene expression profiles or histopathological features. We have explored the prevalence of cells with different CD44/CD24 phenotypes within breast cancer subtypes.

**Methods:**

Double-staining immunohistochemistry was used to quantify CD44 and CD24 expression in 240 human breast tumors for which information on other tumor markers and clinical characteristics was available. Gene expression data were also accessible for a cohort of the material.

**Results:**

A considerable heterogeneity in CD44 and CD24 expression was seen both between and within tumors. A complete lack of both proteins was evident in 35% of the tumors, while 13% contained cells of more than one of the CD44^+^/CD24^-^, CD44^-^/CD24^+ ^and CD44^+^/CD24^+ ^phenotypes. CD44^+^/CD24^- ^cells were detected in 31% of the tumors, ranging in proportion from only a few to close to 100% of tumor cells. The CD44^+^/CD24^- ^phenotype was most common in the basal-like subgroup – characterized as negative for the estrogen and progesterone receptors as well as for HER2, and as positive for cytokeratin 5/14 and/or epidermal growth factor receptor, and particularly common in BRCA1 hereditary tumors, of which 94% contained CD44^+^/CD24^- ^cells. The CD44^+^/CD24^- ^phenotype was surprisingly scarce in HER2+ tumors, which had a predominantly CD24^+ ^status. A CD44^+^/CD24^- ^gene expression signature was generated, which included CD44 and α_6_-integrin (CD49f) among the top-ranked overexpressed genes.

**Conclusion:**

We demonstrate an association between basal-like and particularly BRCA1 hereditary breast cancer and the presence of CD44^+^/CD24^- ^cells. Not all basal-like tumors and very few HER2+ tumors, however, contain CD44^+^/CD24^- ^cells, emphasizing that a putative tumorigenic ability may not be confined to cells of this phenotype and that other breast cancer stem cell markers remain to be identified.

## Introduction

Human breast cancer is a truly complex disease with a large inter-tumoral and intra-tumoral heterogeneity resulting in highly variable clinical behavior and response to therapy. The maintenance of the heterogeneity of cells within a tumor is not fully understood. Possibly, every cell within a tumor may have a capacity to proliferate and form new tumors, although the likelihood for each cell is very low. Alternatively, only a small subset of cells with distinct characteristics has the capacity to maintain tumor growth.

A population of CD44^+^/CD24^-/low ^cells has been demonstrated to have tumor-initiating properties in breast cancer [1]. This tumorigenic phenotype has been associated with stem cell-like characteristics [2], with enhanced invasive properties [3], with radiation resistance [4] and with distinct genetic profiles suggesting correlation to adverse prognosis [5]. The prevalence of CD44^+^/CD24^-/low ^cells within breast tumors, however, has not been significantly associated with clinical characteristics – although tumors with a higher fraction of CD44^+^/CD24^- ^cells were more commonly found in patients diagnosed with distant metastases [6].

Breast cancers have been classified based on their gene expression profiles into luminal A and B, basal-like, HER2+ and normal breast-like subtypes [7-10]. These subtypes have been associated with diverse tumor characteristics and clinical outcome. The luminal subtypes are associated with expression of the estrogen receptor (ER), while basal-like and normal-like tumors are essentially all ER-negative, as are the majority of HER2+ tumors. Multiple studies have demonstrated basal-like tumors to have a particularly poor prognosis [8,9,11], although it is unclear whether basal-like tumors have a significantly worse clinical outcome than other ER-negative tumors [12]. Immunohistochemical features have been used to characterize basal-like tumors as typically negative for ER, for the progesterone receptor (PgR) and for HER2 but positive for basal cytokeratins (CK5/6/14/17), for epidermal growth factor receptor (EGFR) and/or for c-kit [13-15].

A correlation of the CD44^+^/CD24^-/low ^phenotype to specific breast cancer subtypes has not yet been reported in human breast tumors. In the present article we have determined the expression of CD44 and CD24 in human breast tumors using double-staining immunohistochemistry and have correlated the presence of CD44^+^/CD24^- ^cells to subgroups of breast cancer, classified using the expression of ER, PgR, HER2, CK5/14 and EGFR, as well as by mRNA expression profiles. We demonstrate an association of the CD44^+^/CD24^- ^phenotype to basal-like and BRCA1 hereditary breast cancer.

## Materials and methods

### Patients

We studied 240 tumors from a cohort of 445 patients surgically treated for stage II breast cancer (age 31 to 81 years), diagnosed in the South Swedish Health Care Region between 1985 and 1994 and originally participating in two randomized clinical trials [16,17]. All patients received 2 years of adjuvant tamoxifen treatment, without stratification according to ER status. The median follow-up time for patients alive and free from metastasis at the last follow-up visit was 5.3 years. The current study was approved by the Lund University Medical Ethics Committee.

### Tumor characteristics

Fresh-frozen tumor tissue was used for routine determination of cytosolic ER and PgR, as well as the S-phase fraction, using an enzyme immunoassay and DNA flow cytometry, respectively, as described earlier [18,19]. Cores of 0.6 mm diameter formalin-fixed, paraffin-embedded tumor tissue were used to generate tissue microarrays (TMAs) for the 445 cases. Three cores from every individual tumor were arrayed. These TMAs have been used for immunohistochemical staining of CK5, CK14, EGFR and cytokeratin clone AE1/AE3 as described previously [12,20]. A pathologist re-evaluated the histological type on whole formalin-fixed paraffin-embedded sections [21].

### BRCA1 tissue microarray

An additional TMA consisting of tumors from 23 *BRCA1 *germline mutation carriers, diagnosed in Sweden between 1980 and 2001, was used for evaluation purpose. This TMA was generated as described above, including two or three cores from each tumor.

### Immunohistochemical staining

Sections (4 μm) of the TMA blocks were mounted on Dako REAL™ Capillary Gap Microscope Slides (DAKO, Glostrup, Denmark), were deparaffinized in xylene and were rehydrated in ethanol. Antigen retrieval was achieved either by placing slides in Tris-ethylenediamine tetraacetic acid buffer (pH 9.0) at 125°C in a 2100 Retriever (PickCell Laboratories, Amsterdam, the Netherlands) for 5 minutes (CD44/CD24), or by microwaving the slides in Tris-ethylenediamine tetraacetic acid buffer (pH 9.0) for 7 minutes at 800 W followed by 15 minutes at 350 W (HER2).

Double-immunostaining with antibodies for detection of CD44 and CD24 was performed by an Autostainer (DAKO) using EnVision G|2 Doublestain System Rabbit/Mouse (DAB^+^/Permanent Red) (DAKO) according to the manufacturer's instructions. Antibodies for the detection of CD44 (Clone 156-3C11, 1:800) and CD24 (Clone SN3b, 1:400) were purchased from Neomarkers (Fremont, CA, USA). CD44 was detected with Permanent Red and CD24 was detected using diaminobenzidene (DAB).

HER2 was detected with a rabbit polyclonal primary antibody (A0485, 1:1,000; DAKO) followed by EnVision™ on a TechMate™ (DAKO). All slides were counterstained with hematoxylin for the identification of nuclei.

### Immunohistochemical evaluation

The scoring was performed twice by one person in a blinded fashion. All unclear cases were discussed with a pathologist. In case of discrepant staining between the three cores from the same patient, an average was used.

HER2 scoring was carried out according to the standard procedure (DAKO): 0, < 10% of the tumor cells showed membranous staining; 1, > 10% of the tumor cells were positive but not circumferential; 2, weak staining around the whole membrane in > 10% of the tumor cells; and 3, strong staining around the membrane in > 10% of the tumor cells. CD44 staining was detected mainly in the membrane and the scoring was as follows: 0, 0% positive tumor cells; 1, 1% to 10% positive cells; 2, 11% to 50% positive cells; 3, 51% to 75% positive cells; and 4, 76% to 100% positive cells. CD24 staining was detected mainly in the cytoplasm and the scoring was performed as described for CD44.

The proportion of CD44^+^/CD24^- ^tumor cells was determined as the percentage of cells positive for Permanent Red staining but negative for DAB staining. The frequencies of CD44^-^/CD24^+ ^cells and of CD44^+^/CD24^+ ^cells were determined in a similar fashion.

### Statistical analysis

Associations between the presence of CD44, CD24 or different CD44/CD24 phenotypes and clinical variables as well as breast cancer subgroups were assessed by Fisher's exact test, except for age where the Mann-Whitney U test was used. The Kaplan-Meier method was used to estimate distant disease-free survival, and the log-rank test to compare survival between two strata. All tests were two-sided and *P *< 0.05 was considered significant. Statistical analyses were carried out using Stata 10.0 software (Stata Corporation, College Station, TX, USA).

### Microarrays and data analysis

For 168 of the 445 tumors in our cohort, mRNA expression analysis has previously been performed using cDNA microarrays with 27,648 reporters [22,23]. The microarray data for these 168 tumors are available through the Gene Expression Omnibus database (accession numbers GSE6577 and GSE5325). Data pre-processing and filtering for the selected 168 tumors were performed using the BioArray Software Environment [24] as previously described [23], leaving 15,040 reporters that were used for all subsequent analyses.

Three independent sets of gene signatures were used to further characterize the tumors. Genes were matched across datasets based on gene symbols, and matched genes were centralized in our dataset across the 168 tumors. Nearest centroid classifiers were used for the Sørlie and colleagues' [9] and the Hu and colleagues' [10] subtype classifications, with each tumor classified based on to which centroid it was most correlated using Pearson correlation. The average expression level for the genes in a signature was used to characterize tumors for the Shipitsin and colleagues' gene signatures [5].

Hierarchical clustering was performed using MeV in the TM4 system [25] with complete linkage, Pearson correlation distance and gene centralization. Genes with different expression levels in tumors containing CD44^+^/CD24^- ^cells and tumors lacking cells with this phenotype were identified using a two-sided *t *test. To account for multiple comparisons, the false discovery rate was calculated for gene lists. Sixty-nine of the 168 tumors with gene expression profiles were among the 240 tumors for which CD24 and CD44 immunohistochemical stainings were obtained.

## Results

### Immunohistochemical expression of CD44 and CD24

We analyzed the presence of CD44 and CD24 antigens on human breast cancer tissues using double-staining immunohistochemistry. The CD44 and CD24 expression was successfully determined in 240 cases after excluding tumors with scarce tissue on the TMA. These 240 tumors did not significantly differ from the excluded 205 tumors in regard to tumor size, lymph node status, S-phase fraction, ER status or disease-free survival. The median age was slightly higher in the included patients (64 years versus 62 years for excluded patients), although not reaching significance.

**Figure 1:**
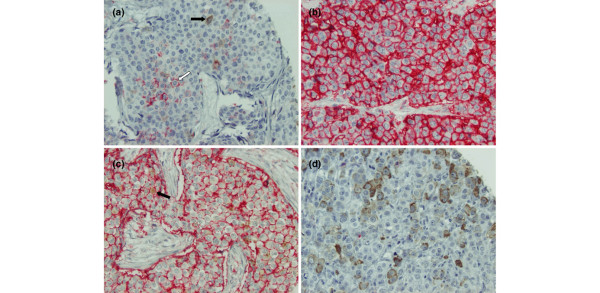
Immunohistochemical double-staining of human breast tumors for CD44 and CD24. CD44 is stained with Permanent Red and CD24 with diaminobenzidene (DAB). Magnification × 20. **(a) **A tumor positive for both CD44^+^/CD24^- ^(white arrow) and CD44^-^/CD24^+ ^(black arrow) cancer cells, although the predominant phenotype is CD44^-^/CD24^-^. **(b) **Almost all cells in this tumor are CD44^+^/CD24^-^. No CD24 staining is seen. **(c) **A tumor with predominantly CD44^+^/CD24^- ^cells. A few CD44^+^/CD24^+ ^cells are also present (black arrow). **(d) **A tumor positive for the CD44^-^/CD24^+ ^phenotype. No CD44 staining is present.

Figure 1 displays representative staining patterns of various breast tumors. Tumor cells were distinguishable from stromal cells and inflammatory cells (generally CD44^+ ^and CD24^-^) by expression of cytokeratin clone AE1/AE3 in adjacent sections. The frequency of tumors with varying degrees of CD44^+ ^and CD24^+ ^tumor cells is presented in Table 1. CD44 showed mainly membranous staining (Permanent Red) with only six tumors displaying cytoplasmic staining, four of which had also membrane staining. Thirty-two percent (77/240) of the tumors had ≥ 1% cells with membranous and/or cytoplasmic CD44 expression and were considered CD44^+^. CD24 was almost exclusively detected in the cytoplasm, with only four tumors displaying membrane DAB staining (all four with positive cytoplasmic staining as well). Forty-six percent (110/240) of the tumors had ≥ 1% cells with CD24 staining and were considered CD24^+^.

**Table 1:** Scoring frequency of different CD44/CD24 phenotypes as determined by immunohistochemistry

Score	CD44 (*n *= 240)	CD24 (*n *= 240)	CD44^+^/CD24^- ^(*n *= 240)	CD44^-^/CD24^+ ^(*n *= 240)	CD44^+^/CD24^+ ^(*n *= 240)
0 (0%)	163 (68)	130 (54)	165 (69)	132 (55)	225 (94)
1 (1% to 10%)	26 (11)	73 (30)	25 (10)	72 (30)	14 (6)
2 (11% to 50%)	22 (9)	20 (8)	22 (9)	20 (8)	0 (0)
3 (51% to 75%)	15 (6)	10 (4)	15 (6)	10 (4)	0 (0)
4 (> 75%)	14 (6)	7 (3)	13 (5)	6 (3)	1 (0.4)

Data presented as *n *(%).

To determine the proportion of putative tumorigenic CD44^+^/CD24^- ^cells within each tumor, we scanned for the presence of Permanent Red staining without any DAB interference. CD44^+^/CD24^- ^tumor cells were detected in 31% (75/240) of the tumors, with the proportion of this phenotype ranging from only a few cells to almost all tumor cells (Figure 1a to 1c). The frequency of tumors with different proportions of CD44^+^/CD24^- ^tumor cells is presented in Table 1. We also determined the proportion of CD44^-^/CD24^+ ^and double-positive (CD44^+^/CD24^+^) cells in each tumor (Table 1). CD44^+^/CD24^+ ^cells (Figure 1c) were detected in 6% (15/240) of the tumors, while CD44^-^/CD24^+ ^cells (Figures 1a, d) were detected in 45% (108/240) of tumors. The majority of tumors included at least a few double-negative (CD44^-^/CD24^-^) tumor cells, while a complete lack of both proteins was evident in 35% (83/240). Thirteen percent (30/240) of the tumors were positive for more than one of the CD44^+^/CD24^-^, CD44^-^/CD24^+ ^and CD44^+^/CD24^+ ^phenotypes, with 5% (11/240) displaying tumor cells of all three phenotypes. In 12% (28/240) of the tumors, the majority (> 50%) of tumor cells was CD44^+^/CD24^-^.

### Correlation of CD44/CD24 status with tumor characteristics

HER2 expression was successfully determined in all 240 cases with CD44/CD24 data, and 16% (38/240) were considered strongly positive (score = 3). Information on other clinical variables and patient survival was available from previous studies. The CD44^+^/CD24^- ^status was significantly correlated to biological characteristics such as low/negative HER2 expression (*P *= 0.002), elevated expression of CK5/14 (*P *= 0.012) and EGFR (*P *= 0.007) (Table 2). Interestingly, all medullary tumors (*n *= 8) were positive for the CD44^+^/CD24^- ^phenotype. The presence of CD44^+^/CD24^- ^tumor cells was not associated with ER and PgR status, with S-phase fraction or with tumor size, nor with lymph node status, menopausal status or age, although showing a tendency of being more common in premenopausal women. Using higher cutoff criteria for the CD44^+^/CD24^- ^status (10% or 50%) and comparing these 50 tumors or 28 tumors, respectively, with all other tumors gave more or less comparable results, although overall slightly higher *P *values (data not shown). The only additional significant association was with ER-negative status using a 10% cutoff value for the CD44^+^/CD24^- ^status (*P *= 0.041).

**Table 2:** Association of CD44^+^/CD24^- ^phenotype with clinical characteristics

Characteristic	All tumors (*n *= 240)	Negative (0%) (*n *= 165)	Positive (> 0%) (*n *= 75)	*P *value^a^
Median age (years)	64.1	64.3	63.5	1.0
Menopausal status				0.098
Premenopausal	42 (18)	24 (15)	18 (24)	
Postmenopausal	198 (83)	141 (85)	57 (76)	
Tumor size				0.58
> 20 mm	183 (76)	143 (75)	40 (80)	
≤ 20 mm	57 (24)	47 (25)	10 (20)	
Lymph node status				0.19
Positive (*n *> 0)	159 (66)	114 (69)	45 (60)	
Negative (*n *= 0)	81 (34)	51 (31)	30 (40)	
S-phase fraction				1.0
High (> 12%)	37 (26)	29 (26)	8 (27)	
Low (< 12%)	106 (74)	84 (74)	22 (73)	
Missing	97			
Histologic type				< 0.001
Ductal	176 (77)	119 (77)	57 (78)	
Lobular	28 (12)	23 (15)	5 (7)	
Lobular + ductal	13 (6)	11 (7)	2 (3)	
Medullary	8 (4)	0 (0)	8 (11)	
Other	3 (1)	2 (1)	1 (1)	
Missing	12			
Estrogen receptor				0.45
Positive (> 25 fmol/mg)	159 (68)	112 (70)	47 (64)	
Negative	74 (32)	48 (30)	26 (36)	
Missing	7			
Progesterone receptor				0.89
Positive (> 25 fmol/mg)	114 (49)	79 (49)	35 (48)	
Negative	119 (51)	81 (51)	38 (52)	
Missing	7			
HER2				0.002
Strong (score = 3)	38 (16)	34 (21)	4 (5)	
Weak/negative	202 (84)	131 (79)	71 (95)	
Cytokeratin 5/14^b^				0.012
Strong	28 (13)	17 (10)	11 (25)	
Weak/negative	188 (87)	155 (90)	33 (75)	
Missing	24			
Epidermal growth factor receptor^c^				
Strong	27 (11)	12 (7)	15 (20)	
Weak/negative	210 (89)	150 (93)	60 (80)	
Missing	3			
Distant metastasis				1.0
Positive	73 (30)	50 (30)	23 (31)	
Negative	167 (70)	115 (70)	52 (69)	

Data presented as *n *(%). ^a^*P *values were calculated using Fisher's exact test, except for age where the Mann-Whitney U test was used. The missing categories were always excluded. ^b^Scoring as previously described [12]. ^c^Scoring as previously described [20].

The presence of CD44^-^/CD24^+ ^tumor cells was solely associated with strong HER2 staining (*P *= 0.002) and not with any other tumor characteristics. The presence of double-positive (CD44^+^/CD24^+^, *n *= 15) tumor cells was not associated with any tumor features, although an increase of tumors of medullary type was indicated compared with tumors lacking cells with this phenotype (23% versus 2%).

### CD44^+^/CD24^- ^status and survival

We did not see any correlation between CD44^+^/CD24^- ^status and distant disease-free survival, nor between CD44^+^/CD24^- ^status and site of distant recurrence. Factors significantly correlated to favorable distant disease-free survival at 5-year follow-up (in this cohort of patients receiving adjuvant tamoxifen therapy) included positive ER and PgR status (*P *= 0.005 and *P *= 0.037, respectively) and negative CK5/14 and EGFR status (*P *= 0.035 and *P *= 0.005, respectively), but not HER2 and lymph node status, while tumor size and a high S-phase fraction reached marginal significance. Lymph node status, however, was correlated to distant disease-free survival in the entire cohort of 445 tumors (*P *= 0.005).

### Definition of breast cancer subgroups by tumor markers and correlation with gene expression subtypes

The CD44^+^/CD24^- ^phenotype was clearly related to certain tumor biological characteristics. To study this relation in more depth, we used five protein markers available for 232 out of the 240 cases with CD44/CD24 data to define five tumor subgroups. Tumors positive for ER and/or PgR were designated steroid receptor positive (SR+). The SR and HER2 status was used to broadly divide tumors into four subgroups; SR+HER2- (*n *= 150), SR+HER2+ (*n *= 14), SR-HER2+ (*n *= 24), and SR-HER2- (triple negative, *n *= 44). The latter group was further subdivided into a subgroup expressing basal CK5/14 and/or EGFR (SR-HER2- basal-like, *n *= 30), and a subgroup negative for all five markers (SR-HER2- nonbasal, *n *= 14).

**Figure 2:**
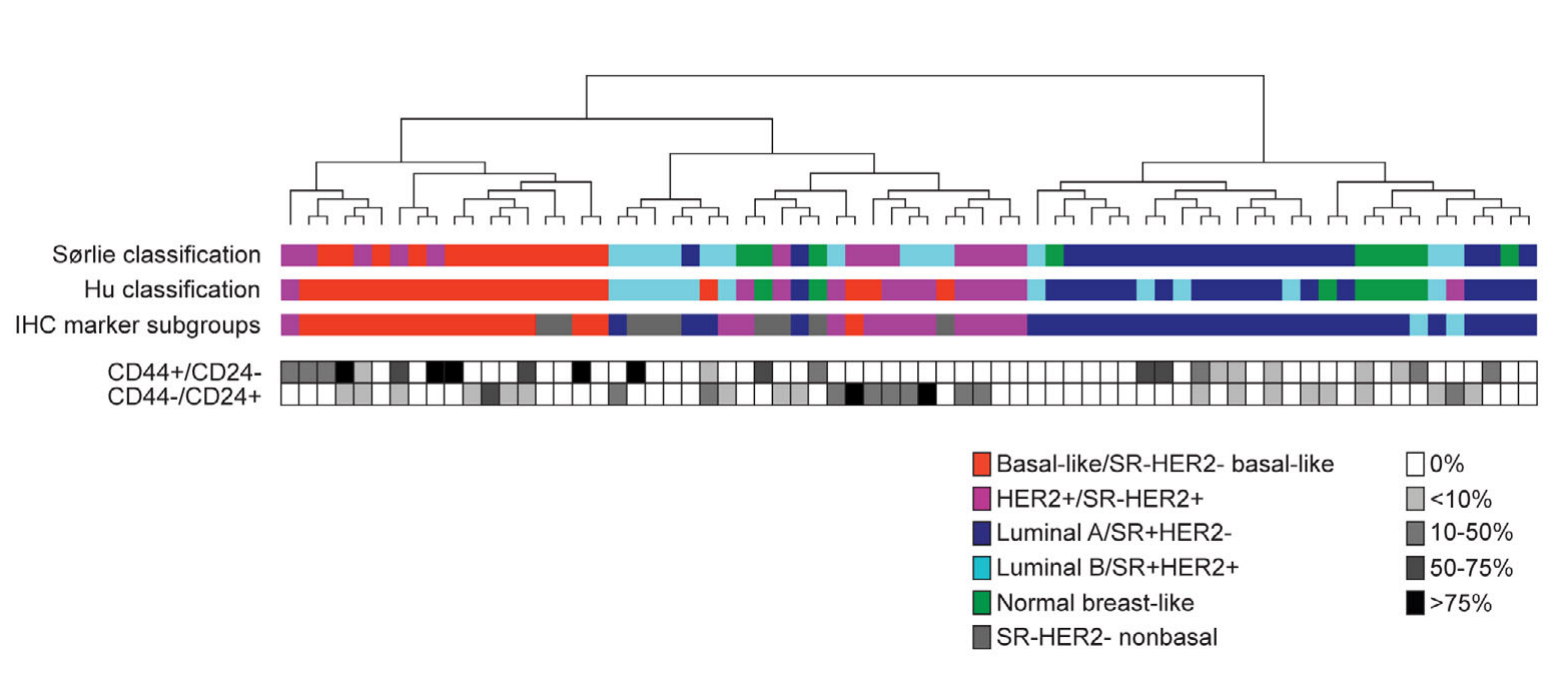
Hierarchical clustering of 69 tumor samples with available gene expression data. Clustering was based on 364 genes from the intrinsic gene list published by Sørlie and colleagues [9] that matched our cDNA clones. Colored boxes indicate classification of each tumor into subtypes/subgroups. Filled or open boxes indicate the percentage of cells in each tumor positive for the CD44^+^/CD24^- ^and CD44^-^/CD24^+ ^phenotypes as determined by immunohistochemistry. SR, steroid receptor. Hu classification, Hu and colleagues [10].

Gene expression data were available for 69 of the 232 tumors, which allowed us to correlate our five subgroups, as defined by the five tumor markers, with breast cancer subtypes defined by gene expression profiling and intrinsic gene lists [9,10]. As seen in Figure 2, SR+HER2- tumors were clearly enriched for luminal A tumors, while SR-HER2- basal-like tumors corresponded prominently to the basal-like subtype defined by gene expression profiling. As expected, SR-HER2+ tumors correlated well with the HER2+ subtype. The SR-HER2- nonbasal subgroup showed no clear association with any tumor subtype – and only two tumors were SR+HER2+, making it difficult to draw any conclusions for this subgroup. We therefore conclude that tumor classification based on a combination of five commonly used tumor protein markers is biologically relevant for subgroup analysis in our tumor material.

### CD44/CD24 status in different breast cancer subgroups

The expression of CD44 and CD24 differed significantly between the subgroups (*P *= 0.001 and *P *= 0.035, respectively) (Table 3). CD44 was highly expressed in the SR-HER2- basal-like subgroup, with 63% of the tumors being positive compared with 32% for the entire cohort, and was very lowly expressed in the HER2+ groups, with only 14% and 17% of the tumors being positive in the SR+HER2+ and SR-HER2+ groups, respectively. CD24 was highly expressed in the SR-HER2+ group (75% compared with 47% for the entire cohort).

**Table 3:** Association of CD44/CD24 phenotypes with breast cancer subgroups.

Phenotype	All tumors (*n *= 232)	SR+HER2- (*n *= 150)	SR+HER2+ (*n *= 14)	SR-HER2+ (*n *= 24)	SR-HER2-		*P *value^a^
						
					Basal-like (*n *= 30)	Nonbasal (*n *= 14)	
CD44							0.001
Positive (> 0%)	75 (32)	47 (31)	2 (14)	4 (17)	19 (63)	3 (21)	
Negative	157 (68)	103 (69)	12 (86)	20 (83)	11 (37)	11 (78)	
CD24							0.035
Positive (> 0%)	108 (47)	65 (43)	8 (57)	18 (75)	12 (40)	5 (36)	
Negative	124 (53)	85 (57)	6 (43)	6 (25)	18 (60)	9 (64)	
CD44^+^/CD24^-^							< 0.001
Positive (> 0%)	73 (31)	47 (31)	2 (14)	2 (8)	19 (63)	3 (21)	
Negative	159 (69)	103 (69)	12 (86)	22 (92)	11 (37)	11 (79)	
CD44^-^/CD24^+^							0.027
Positive (> 0%)	106 (46)	63 (42)	8 (57)	18 (75)	12 (40)	5 (36)	
Negative	126 (54)	87 (58)	6 (43)	6 (25)	18 (60)	9 (64)	
CD44^+^/CD24^+^							0.61
Positive (> 0%)	14 (6)	9 (6)	0 (0)	3 (12)	2 (7)	0 (0)	
Negative	218 (94)	141 (94)	14 (100)	21 (88)	28 (93)	14 (100)	
CD44^-^/CD24^-^							0.28
100%	78 (28)	57 (31)	4 (24)	5 (18)	6 (18)	6 (40)	
< 100%	197 (72)	125 (69)	13 (76)	23 (82)	27 (82)	9 (60)	

Data presented as *n *(%). SR, steroid receptor. ^a^*P *values were calculated using Fisher's exact test.

The frequency of tumors positive for the CD44^+^/CD24^- ^phenotype varied significantly between the different subgroups (*P *< 0.001, Table 3). While close to two-thirds (63%) of tumors resembling the basal-like subtype (SR-HER2- basal-like) expressed CD44^+^/CD24^- ^cells, this phenotype was very low in HER2+ tumors, regardless of SR status: 8% in SR-HER2+ tumors and 14% in SR+HER2+ tumors (Table 3). The divergence between subgroups remained when the cutoff level for CD44^+^/CD24^- ^was raised to 10% or 50% (*P *= 0.003 and *P *= 0.033, respectively). The frequency of CD44^+^/CD24^- ^cells within tumors positive for this phenotype was also higher in basal-like tumors, as indicated in Figure 2 for the 69 tumors with gene expression data.

The proportion of tumors with CD44^-^/CD24^+ ^cells also differed significantly between subgroups (*P *= 0.027), with the highest proportion in the two HER2+ groups (SR-HER2+ and SR+HER2+) (Table 3). The presence of double-positive (CD44^+^/CD24^+^) and double-negative (CD44^-^/CD24^-^) tumor cells did not differ between subgroups (Table 3).

### CD44^+^/CD24^- ^status in BRCA1-defective tumors

Since there was a clear correlation between the CD44^+^/CD24^- ^status and the basal-like tumor subtype, we extended the immunohistochemical analysis to an additional TMA including tumors from BRCA1 hereditary breast cancer patients, known to be of predominantly basal-like phenotype [9]. A basal-like status was verified for nine of our BRCA1 hereditary tumors for which gene expression data were available.

Seventeen of the 23 BRCA1-defective tumors were successfully stained for CD44 and CD24 expression. The frequency of tumors with different proportions of CD44^+^/CD24^- ^cells is presented in Table 4. Ninety-four percent (16/17) of the tumors were positive for this phenotype, thus corroborating the finding of a high proportion of CD44^+^/CD24^- ^cells in the SR-HER2- basal-like subgroup (Table 4). *BRCA1 *germline mutant tumors, however, also had a high proportion (70%) of cells positive for the CD44^-^/CD24^+ ^phenotype, considerably higher than the 40% seen among SR-HER2- basal-like tumors.

**Table 4:** Scoring frequency of CD44^+^/CD24^- ^cells in BRCA1-defective breast tumors in comparison with SR-HER2- basal-like tumors

Score	SR-HER2- basal-like (*n *= 30)	BRCA1 mutant (*n *= 17)
0 (0%)	11 (37)	1 (6)
1 (1% to 10%)	4 (13)	4 (24)
2 (11% to 50%)	6 (20)	6 (35)
3 (51% to 75%)	4 (13)	1 (6)
4 (> 75%)	5 (17)	5 (29)

Data presented as *n *(%). SR, steroid receptor.

### Correlation to published prognostic gene signatures

We correlated the presence of CD44^+^/CD24^- ^cells to two gene signatures with prognostic value specific for either CD44^+ ^or CD24^+ ^breast cancer cells published by Shipitsin and colleagues [5]. Signatures A and B are associated with shorter and longer distant recurrence-free survival time, respectively. A positive correlation of CD44^+^/CD24^- ^cells to signature A was seen if the cutoff level for CD44^+^/CD24^- ^was 50% or 75% positive cells (*P *= 0.05 and *P *= 0.008, respectively) but not when using lower cutoff levels. A negative correlation to signature B was seen when using cutoff levels of 0%, 10% or 75% positive cells (*P *< 0.001, *P *< 0.001 and *P *= 0.01, respectively).

### Gene expression signature of the CD44^+^/CD24^- ^phenotype

To identify a gene expression signature for the CD44^+^/CD24^- ^phenotype, we used the 69 tumors with gene expression data and looked for genes differentially expressed between tumors containing CD44^+^/CD24^- ^cells and tumors lacking such cells. The top 20 genes are displayed in Table 5. Interestingly, CD44 emerged as the second ranked gene, demonstrating a good correspondence between protein and mRNA levels for this gene – although the top 20 genes collectively had a relatively high false discovery rate of 28%.

**Table 5:** Top 20 genes differing between tumor with and without the presence of CD44^+^/CD24^- ^tumor cells

Rank	Clone^a^	Gene description^b^	Gene symbol	Sign^c^
1	1499830	Myocardial infarction associated transcript (nonprotein coding)	MIAT	-
2	82421	CD44 molecule (Indian blood group)	CD44	+
3	2018758	Peroxisomal biogenesis factor 7	PEX7	+
4	320712	Protein kinase, X-linked, pseudogene 1	PRKXP1	-
5	824908	Transmembrane protein 49	TMEM49	-
6	328868	CD44 molecule (Indian blood group)	CD44	+
7	1472689	Apolipoprotein C-I	APOC1	-
8	126341	Myosin, light chain kinase	MYLK	+
9	1609752	Apolipoprotein C-I	APOC1	-
10	811028	Transmembrane protein 49	TMEM49	-
11	788714	Ataxin 10	ATXN10	+
12	310019	Myosin, light chain kinase	MYLK	+
13	137296	SEC24 related gene family, member A (*S. cerevisiae*)	SEC24A	-
14	32493	Integrin, α_6_	ITGA6	+
15	754582	Ecotropic viral integration site 2A	EVI2A	-
16	1159963	Interferon regulatory factor 7	IRF7	-
17	612576	3-Oxoacid CoA transferase 1	OXCT1	+
18	503724	Transcribed locus, strongly similar to NP_034722.1 jun D proto-oncogene (*Mus musculus*)	Unavailable data	+
19	810463	Dehydrogenase/reductase (SDR family) member 7B	DHRS7B	-
20	502067	Lupus brain antigen 1	LBA1	-

^a^Integrated Molecular Analysis of Genomes and their Expression (IMAGE) consortium cDNA clone number. ^b^Clones mapped using UniGene Build 208 (National Center for Biotechnology Information, Bethesda, Maryland, USA). ^c^Genes are either upregulated (+) or downregulated (-) in tumors containing CD44^+^/CD24^- ^tumor cells.

## Discussion

The concept of cancer stem cells relies on the presence of a subpopulation of cells within tumors that drives tumorigenesis, as well as giving rise to a large population of differentiated progeny that constitute the bulk of the tumor but lack tumorigenic potential [26]. Multiple studies indicate that CD44^+^/CD24^- ^breast cancer cells have tumor-initiating properties [1-3].

In the present study, we have explored the dual expression of CD44 and CD24 in a sample of 240 stage II breast tumors with specific regard to breast cancer subtypes. The CD44 staining was almost exclusively membranous, which is concordant with prior literature [27], while CD24 predominantly stained the cytoplasm. Earlier publications have shown either membranous and/or cytoplasmic CD24 staining [28,29]. Bircan and colleagues observed a cytoplasmic CD24 staining pattern in neoplastic breast tissue while it was mainly detected in the cell membrane in normal breast [30]. Intracytoplasmic CD24 expression has been suggested to reflect overexpression of the protein or disturbance of the protein distribution or degradation in neoplastic cells [31]. It is reasonable to suspect that cells with a cytoplasmic CD24 pattern also express CD24 protein on the cell surface. The specificity of the CD24 antibody has earlier been ascertained by flow cytometric analysis [32].

Overall, we saw a large heterogeneity of CD44 and CD24 expression between tumors, but also within tumors where the proportion of positive cells varied considerably. Interestingly, tumor cells were mostly positive for either CD44 or CD24 and rather few tumors contained double-positive cells, although it was quite common that individual tumors contained both CD44^+ ^and CD24^+ ^cells. A recent study by Shipitsin and coworkers implicated that CD24^+ ^and CD44^+ ^cells within breast carcinomas represent defined cell populations with distinct genetic profiles [5]. They showed that CD24^+ ^cells were more differentiated while CD44^+ ^cells had more progenitor-like features, suggesting that CD24^+ ^cells might be derived from CD44^+ ^cells. Findings from our study could support this hypothesis, in that the variable presence of tumor cells that are largely either CD44^+ ^or CD24^+ ^may reflect the current state of a tumor undergoing constant cell renewal, differentiation and death at a pace defined by their intrinsic machinery and interaction with surrounding stroma.

Contradictive to results by Al-Hajj and colleagues demonstrating CD44^+^/CD24^- ^cells in all their breast cancer samples [1], we only detected cells with this phenotype in 31% of our tumors. This discordance could depend on their study involving mainly metastatic tissues, including only one primary tumor. Metastatic tumor cells may have a more stem cell-like phenotype, as indicated by enrichment of the CD44^+^/CD24^- ^phenotype in bone marrow micrometastasis of breast cancer patients [33]. A recent study demonstrated CD44^+^/CD24^- ^cells in 59% of human breast tumors, further supporting that not all breast tumors contain cells with this phenotype [34].

We found that the CD44^+^/CD24^- ^status was associated with low/negative HER2 expression and with elevated expression of CK5/14 and EGFR, as well as with medullary histological type, all known characteristics of the basal-like subtype of breast cancer. This motivated further analysis of the prevalence of CD44^+^/CD24^- ^cells in different tumor subtypes.

Since previous studies have demonstrated a good resemblance of subgroups defined by common tumor markers to molecular subtypes defined by mRNA expression patterns [13,14], we used cytosolic protein levels of ER and PgR and used TMA immunostaining of HER2, CK5/14 and EGFR to classify the material into five tumor subgroups. Using available gene expression data for 69 of the tumors and using published intrinsic gene lists [9,10], we could demonstrate a reasonable correlation to molecular subtypes for three of our five subgroups, justifying a subgroup analysis of CD44^+^/CD24^- ^expression. We could thereby demonstrate an association between the presence of CD44^+^/CD24^- ^tumor cells and a basal-like subgroup of breast cancer. This finding is consistent with a recent publication where Sheridan and colleagues observed a correlation between breast cancer cell lines with a basal/myoepithelial origin and CD44^+^/CD24^- ^expression [3].

Moreover, we observed that basal-like tumors often had a higher proportion of CD44^+^/CD24^- ^cells, while tumors of other subtypes that contained CD44^+^/CD24^- ^cells generally had a lower number of cells with this phenotype. This observation corroborates prior work indicating that basal-like tumors have a greater stem cell-like phenotype [35]. These tumors may originate from the most primitive ER-negative stem/progenitor cells, suggesting a block in differentiation upstream of ER-positive progenitor cells [36]. Tumors developing in *BRCA1 *germline mutation carriers are typically of basal-like subtype, possibly due to the critical role BRCA1 plays in the differentiation of ER-negative stem/progenitor cells to ER-positive luminal cells [37]. Accordingly, we found that all but one of the 17 BRCA1 tumors contained CD44^+^/CD24^- ^cells, further illustrating the correlation between CD44^+ ^status and basal-like/BRCA1-like tumors.

Interestingly, the HER2+ tumor subtype, generally considered an aggressive form of breast cancer, displayed a low frequency of tumors containing CD44^+^/CD24^- ^cells and was in fact often positive for the CD44^-^/CD24^+ ^phenotype. CD24 expression has been shown to contribute to the more differentiated state of committed cells [38], but has also been associated with rapid cell spreading, increased cell motility and invasion [39]. In normal breast epithelium, basal/myoepithelial cells but not luminal epithelial cells express CD44 [27] while CD24 is highly expressed in luminal cells [40]. The predominantly CD24^+ ^phenotype of HER2+ tumors may reflect the origin for at least some of these tumors from a CD24^+ ^luminal epithelial cell type [40].

We did not see any association between the CD44^+^/CD24^- ^status and markers known to be important for the clinical outcome, including tumor size, nodal status or S-phase fraction. These results are partly in accordance with previous observations by Abraham and colleagues [6], although these authors reported that tumors with a higher fraction of CD44^+^/CD24^- ^cells were more commonly found in patients diagnosed with distant metastases. We saw no such trend in our material, regardless of whether analyzing the whole material or different subgroups separately.

Since all patients in our study received adjuvant tamoxifen therapy the lack of correlation to survival should however be cautiously interpreted. We therefore evaluated prognostic gene signatures specific for either CD44^+ ^cells (signature A) or CD24^+ ^cells (signature B) published by Shipitsin and colleagues [5], shown to be associated with short and long distant recurrence-free survival, respectively, in patients not undergoing adjuvant systemic therapy. Interestingly, we saw a significant positive correlation of tumors with a high (> 50%) proportion of CD44^+^/CD24^- ^cells to signature A, and a significant negative correlation between the presence of CD44^+^/CD24^- ^cells and signature B. This observation indicates that a high proportion of CD44^+^/CD24^- ^cells could therefore be a marker of aggressive phenotype also in our material, although we saw no correlation with prognosis.

We detected a gene expression signal for tumors containing CD44^+^/CD24^- ^cells. Although the false discovery rate was high, the signature is strengthened by the fact that CD44 occurs as the top second gene. Interestingly, α_6_-integrin also appears as one of the top overexpressed genes. This gene (also known as CD49f) has previously been used to identify mammary epithelial stem cells [41] and was recently demonstrated to be necessary for tumorigenicity of MCF7 breast cancer cells [42].

## Conclusion

Our results demonstrate a clear variation in the prevalence of CD44^+^/CD24^- ^tumor cells between breast tumors of different subtypes. The occurrence of this phenotype is high in basal-like tumors – and especially in BRCA1 hereditary tumors – is lower in tumors of luminal type and is particularly low in the HER2+ tumors, irrespective of ER status. These results emphasize the biological heterogeneity of breast cancer and an enrichment of putative tumor-initiating cells in the aggressive basal-like tumor subtype. Far from all basal-like tumors contain CD44^+^/CD24^- ^cells, however, and their scarcity in HER2+ tumors suggests that tumorigenicity may not be confined to cells of this phenotype and that other markers remain to be identified. Moreover, the obvious heterogeneity of cells with various CD44/CD24 expression within individual tumors may be indicative of a cancer stem cell subpopulation giving rise to more differentiated and committed cell populations. This does by no means exclude the coexistence of cancer cell clones of independent origin, evolution and tumorigenic ability.

## Abbreviations

CK = cytokeratin; DAB = diaminobenzidene; EGFR = epidermal growth factor receptor; ER = estrogen receptor; PgR = progesterone receptor; SR = steroid receptor; TMA = tissue microarray.

## Competing interests

The authors declare that they have no competing interests.

## Authors' contributions

GH participated in the design of the study, evaluated the immunostainings, performed the statistical analysis and drafted the manuscript. P-OB supported the statistical analysis. MR conducted the gene expression classification and supported the draft of the paper. LHS and SKG-S provided gene expression data and comments to the manuscript. KL conducted the immunostainings. DG supported the evaluation of the immunostainings. MF contributed to the design of the study and the interpretation of the results. ÅB participated in the design and coordination of the study and helped to draft the manuscript. CH conceived of the study, participated in its design, performed the microarray analysis and helped to draft the manuscript. All authors read and approved the final manuscript.
